# On the Application of Clustering and Classification Techniques to Analyze Metabolic Syndrome Severity Distribution Area and Critical Factors

**DOI:** 10.3390/ijerph16091575

**Published:** 2019-05-06

**Authors:** Chien-Chih Wang, Jin-Jiang Jhu

**Affiliations:** Department of Industrial Engineering and Management, Ming Chi University of Technology, New Taipei 24301, Taiwan; jinjiang0315@gmail.com

**Keywords:** metabolic syndrome, integrated community health screening, decision trees

## Abstract

In recent years, metabolic syndrome has become one of the leading causes of death in Taiwan. This study proposes a classification and clustering method specific to the administrative regions of New Taipei City to explore the incidence and corresponding risk factors for metabolic syndrome in various geographic areas. We used integrated community health screening data and survey results obtained from people aged ≥40 years in each of the administrative regions of New Taipei City as study samples. Using a combination of Ward’s method, multivariate analysis of variance, and k-means, we identified administrative regions of New Taipei City with metabolic syndrome incidences of a similar nature. Classification and regression tree methods were used to discover the key causes of metabolic syndrome in each region based on lifestyles and dietary habits. The administrative regions were divided into four groups: high-risk, slightly high-risk, normal-risk, and low-risk. The results showed that the severity of metabolic syndrome varies by region and the risk factors for metabolic syndrome vary by region. It has also been found that regions with a higher incidence of metabolic syndrome have relatively fewer medical resources.

## 1. Introduction

Metabolic syndrome is a risk factor for diseases such as cardiovascular diseases, gout, and arthritis. Metabolic syndrome is not a disease itself but rather is a health alert that has a high correlation with cerebrovascular disease, cardiovascular disease, diabetes, nephropathy, and hypertension. According to the 2016 statistics from the Ministry of Health and Welfare, at least three million people in Taiwan have metabolic syndrome. The mortality rate of individuals with this condition is 2.5 times that of those without the condition. The number of deaths due to diseases associated with metabolic syndrome, such as cardiovascular disease, cerebrovascular disease, diabetes, hypertension, and nephropathy, has become higher than those due to cancer [1]. High-calorie, high-fat, and low-fiber food products have become the primary source of food for most people. This change has led to an excessive consumption of calories and fat, causing an imbalance in nutrient intake and, together with an insufficient amount of exercise, constitute a major cause of metabolic syndrome; it has become the biggest health threat to Taiwan’s population. If effective prevention measures are implemented to lower its incidence, the incidence of related diseases would be reduced, in turn saving medical resources and reducing the burden on the health care system.

Previous studies have found that age is an important factor affecting the incidence of metabolic syndrome. Its incidence appears to show an upward trend with increasing age [2,3]. Differences were also found in incidence rates between races and sexes. A study by Meigs et al. [4] using the National Cholesterol Education Program (NCEP) Adult Treatment Panel III (ATP III) guidelines found that the incidence rates of metabolic syndrome in white men and women were 26.9% and 21.4%, respectively, and those in Mexican-American men and women were 29.0% and 32.8%, respectively. Moon et al. [5] used the Euro-American and Asian editions of the NCEP ATP III guidelines to estimate the incidence rates of metabolic syndrome in Korean men and women, which were 29.2% and 19.2%, respectively. Tan et al. [3] used the Asian edition of the NCEP ATP III guidelines to estimate the incidence rates of metabolic syndrome in Singaporean men and women, which were 20.9% and 15.5%, respectively. A study by Lai et al. [6] found that the number of people who smoke who have metabolic syndrome is 1.61 times that of those who do not smoke. A study by Chiu et al. [7] revealed that the number of people who chew betel nut and suffer from metabolic syndrome is 1.86 times that of those who do not. Obesity is also a major cause of metabolic syndrome. A study by Hsieh [8] found that among 11 obese patients with metabolic syndrome, four had fully recovered after 12 weeks of regular training using a treadmill. Ni et al., in a study of spatial statistics in health care, proposed a weighted network kernel density estimation method to identify significant differences between the outside and inside areas of the Ming city wall. They found that the distribution of hospitals correlates highly with the street centralities, and that the correlations are higher with private and small hospitals than with public and large hospitals [9]. Yang et al. proposed the multicriteria evaluation (MCE) analysis model for the location assessment in constructing new hospitals to improve healthcare accessibility in suburban areas of Wuhan [10], and Tao et al. proposed the multi-modal two-step floating catchment area method to improve the estimation of travel time by public transit and by car. The results showed that public transportation conditions for access to medical services had been improved and had reduced the disparity of healthcare accessibility [11].

New Taipei City, with a population of approximately four million, is Taiwan’s most populated and most extensive municipality, with 29 regions under its jurisdiction. It ranks 62nd in the global urban population. Due to significant differences in population structure and lifestyle, there are large demographic and lifestyle variations in the administrative regions, each of which is similar to a small country. In terms of medical resources, the distribution is nonuniform for each region. Among 3011 home-care hospitals in New Taipei City, 2026 (67.28%) hospitals are concentrated in the few highly urbanized regions, such as Banqiao, Zhonghe, Sanchong, Xinzhuang, Yonghe, and Xindian. The remaining regions rely on just a few clinics to provide medical services, of which the eight regions of Shiding, Pinglin, Shimen, Pingxi, Shuangxi, Gongliao, Wanli, and Wulai have only 26 clinics. Therefore, if we could understand the disease patterns in each administrative region, major health issues in different administrative regions could be identified. In terms of public health, there could be a more effective distribution of resources, and public health strategies could be tailored to the administrative regions.

Since 2003, New Taipei City has promoted an integrated community screening plan in an effort to promote preventive health care services in each region. The screening includes adult health examinations, elderly health examinations, Pap tests, fecal colorectal cancer screening, and mammography screening. The targets for these services include citizens of New Taipei City between the ages of 40–64 years, people who have not undergone adult health examinations in the last 3 years, and those aged >65 years who have not undergone elderly health examinations in the current year. We analyzed the integrated community health screening data obtained from people aged ≥40 years in each of the administrative regions of New Taipei City, and applied clustering technology to perform optimized grouping to evaluate the incidence rates of five risk factors for metabolic syndrome and to find regions with similar incidence rates. Then, decision trees were used to perform further analyses specific to each group based on the degree of risk, with the aim of identifying differences in lifestyles and dietary habits in the administrative regions and developing corresponding public health strategies. We hope that this will lead to a more effective and efficient distribution of medical resources and lower the incidence of metabolic syndrome.

## 2. Materials and Methods

We analyzed the integrated community health screening data obtained from each of the administrative regions of New Taipei City. First, metabolic syndrome was defined, and incidence rates were calculated. Then, statistical analyses were performed on the incidence rates and abnormal distributions of metabolic syndrome for each region. Subsequently, clustering technology was used to group the regions based on the severity of metabolic syndrome. Finally, with the information on the population’s demographics, lifestyle, and dietary habits obtained from the integrated community health screening survey, decision trees were used to determine the corresponding risk factors for each group.

We used risk factors for metabolic syndrome, including abdominal obesity, high blood pressure, high fasting blood glucose level, high triglyceride level, and low high-density lipoprotein cholesterol level, as determined by the Health Promotion Administration of the Ministry of Health and Welfare (Table 1). Metabolic syndrome was defined as the presence of three or more of the abovementioned risk factors. Prevalence rates refer to the number of people with metabolic syndrome in proportion to the total population at a given time or within a given period of time. It is defined as follows:

(1) $$P_{x}\left(t\right)=\frac{S_{x}\left(t\right)}{I_{x}\left(t\right)}$$

$S_{x}\left(t\right)$ indicates the number of people with metabolic syndrome in the *x*-year-old population at *t* years; $I_{x}\left(t\right)$ indicates the total number of people in the *x*-year-old population at *t* years.

### 2.1. Clustering Technology

We employed a combination of Ward’s method, multivariate analysis of variance (MANOVA), and k-means to determine the administrative regions with metabolic syndrome of similar characteristics in New Taipei City. Suppose there are *k* administrative regions and each administrative region has *i* number of incidence variables. Ward’s grouping procedure is as follows:

- Step 1: First, set each administrative region as a group, starting with *k* groups.
- Step 2: Perform the first merge by merging *k* groups into one *k* − 1 group.
- Step 3: Continue Step 2 by continuing to merge.
- Step 4: Construct a tree diagram for each stage of grouping, with the horizontal axis as the administrative regions and the vertical axis as the distance between the groups.
- Step 5: Observe the tree diagrams and obtain preliminary grouping numbers.

Previous studies have used empirical methods to determine the grouping of tree diagrams. This study proposes using MANOVA, with the grouping result as the dependent variable and the incidence rate as the independent variable to evaluate the groupings in order to confirm the largest difference between the groups. The criteria for determination are the possible groupings obtained from Step 4, or approximately 2 to 3 groupings. Then, MANOVA was performed, and the *p*-value was calculated. A *p*-value of <0.05 indicated the grouping to be appropriate. If there was one grouping only during Step 4, then grouping was stopped; otherwise, Pillai’s trace index was calculated. The largest Pillai’s trace index was chosen as the best grouping for Step 4.

Sometimes, because of the results obtained from Ward’s method some regions will be put in a group, and these regions will always remain in that group. This study includes the k-means method to compensate for this deficiency. The best groupings obtained from the Ward’s method and MANOVA were used as a basis for performing k-means. The calculation procedures for k-means are as follows:

- Step 1: Choose a starting core point: Initialize the core point, and start with the *n* total number of points $\left\{x_{1},x_{2},\ldots ,x_{n}\right\}$. Select the *k* number of core points $\left\{z_{1},z_{2},\ldots ,z_{k}\right\}$ from that total.
- Step 2: Grouping action: Compare the *k* number of core points $\left\{z_{1},z_{2},\ldots ,z_{k}\right\}$ according to the *n* total number of points. If the distance between $x_{i}$ and $z_{j}$ is the smallest, then $x_{i}$ belongs in group *j*. Continue this step until every point belongs to a group.
- Step 3: Recalculating new core points: Calculate new core points $\left\{z_{1}^{*},z_{2}^{*},\ldots ,z_{k}^{*}\right\}$ using the following equation:
  (2) $$z_{i}^{*}=\underset{d_{i}\in S_{k}}{\sum }\frac{d_{i}}{\left|S_{k}\right|}$$
  where $\left|s_{k}\right|$ is the data belonging in group *k*.
- Step 4: Condition for stopping grouping: Repeat Steps 2 and 3 until there are no more variations in the core points.

### 2.2. Classification Technology

For regions with variations in the severity of metabolic syndrome, the classification and regression tree (CART), a type of decision tree analysis was used to identify key causes specific to the data collected [12]. The analysis steps were as follows:

- Step 1: First, pick a rule *i* and perform segmentation.
- Step 2: Calculate $P_{L}$ and $P_{R}$. $P_{L}$ represents the probability of matching the total sample before division in Step 1, and $P_{R}$ represents the probability of not matching the total sample before division in Step 1.
- Step 3: Calculate the impurity function value under the rules of Step 1. The definition of impurity function is as follows:
  (3) $$i\left(t_{i}\right)=-\overset{n}{\underset{i}{\sum }}P(t_{i}|t_{0})\times logP(t_{i}|t_{0})$$
  where $t_{i}$ represents the two groups divided under a single rule, including $t_{L}$ and $t_{R}$.
- Step 4: Calculate Δi(Δt) using the following equation: $\Delta i\left(\Delta t\right)=i\left(t\right)-P_{L}i\left(t_{L}\right)-P_{R}i\left(t_{R}\right)$. If Δi(Δt)>0, then proceed to Step 5; if Δi(Δt)<0, then skip to Step 6.
- Step 5: Store the rule and Δi(Δt) value from Step 1, and find the next node. If a node exists, then return to Step 1. If not, then proceed to Step 7.
- Step 6: Do not store the rule and Δi(Δt) value from Step 1, and find the next node. If a node exists, then return to Step 1. If not, then proceed to Step 7.
- Step 7: Perform sequencing for all Δi(Δt) values stored previously, and find the largest Δi(Δt).
- Step 8: Use the rule *i* segmented by the largest Δi(Δt) to perform segmentation, and generate new nodes.
- Step 9: After achievement of the sample number $n_{t}$ preset by the terminal node, stop the process. After stopping, appoint each node’s majority class as that node’s final class. If the stopping condition is not achieved, return to Step 1.

## 3. Results 

We used integrated community health screening data from the Department of Health, New Taipei City, from 2015. The database included data from 29 administrative areas in New Taipei City and a total screening population of 28,304. After the deduction of 3326 screenings and lost surveys, 24,978 valid samples were obtained. 

**Figure 1:**
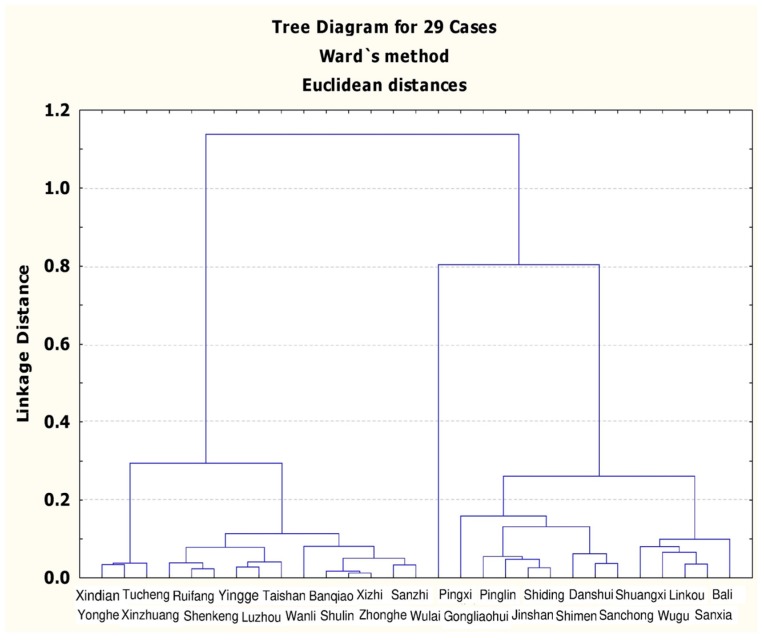
Tree diagram for the results of Ward’s grouping method.

Using the standard of determination for metabolic syndrome, the screening data were divided into groups, with 4886 people having no disease, 6529 with one disease, 5913 with two diseases, 4339 with three diseases, 2468 with four diseases, and 843 with five diseases. The prevalence rate of metabolic syndrome was 0.8, 95% CI 0.799–0.809. Additionally, the prevalence analysis showed that the incidence rate for people aged >40 years with metabolic syndrome was 30.63%, whereas for those aged >65 years it was 42.23%. The incidence rate for men was 33.72%, higher than the 28.76% found for women. In terms of individual age group and sex, the incidence rates were as follows: women aged >65 years (47.03%); men aged >65 years (36.96%); men aged 40–64 years (32.67%); and women aged 40–64 years (25.21%). Based on the calculated incidence rate, Figure 1 shows the results of Ward’s grouping method.

From Figure 1, groups 2 to 4 can be distinguished, and then MANOVA was employed to calculate the group numbers (Table 2). When the data in Figure 1 were divided into groups 2 to 4, the *p*-values were all lower than 0.05, achieving a significance level and satisfying the demand for a reasonable number of groups. After calculating the Pillai’s trace for groups 2 to 4, it was found that the Pillai’s trace value was the largest when the number of groups was four; therefore, the best number of groups in this stage was four.

**Figure 2:**
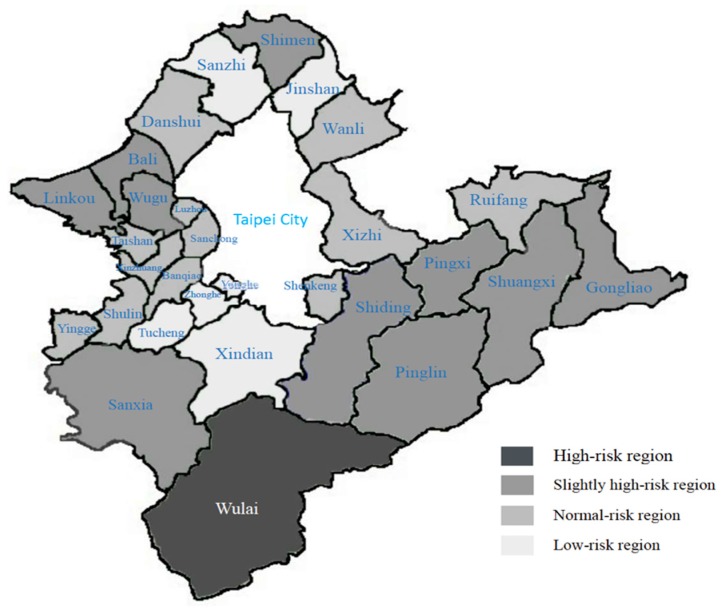
Distribution of severity of metabolic syndrome in New Taipei City.

As Figure 2 shows, the k-means were calculated to fine-tune the individual points in each group, dividing the results into high-risk, slightly high-risk, normal-risk, and low-risk regions.

- The high-risk region includes one administrative region, Wulai. The incidence rates of the five risk factors for metabolic syndrome were the highest in this administrative region (high-density lipoprotein cholesterol 50.63%; triglycerides 46.84%; blood sugar 54.43%; blood pressure 73.42%; abdominal circumference 62.03%; and overall incidence rate 64.56%).
- The slightly high-risk regions include ten administrative regions: Shiding, Pinglin, Gongliao, Shimen, Sanxia, Wugu, Pingxi, Shuangxi, Linkou, and Bali. The incidence rates of the five risk factors for metabolic syndrome were 37.87% for high-density lipoprotein cholesterol, 20.91% for triglycerides, 38.79% for blood sugar, 61.00% for blood pressure, and 45.39% for abdominal circumference; the overall incidence rate was 36.63%. Other than the incidence rate for high-density lipoprotein cholesterol in these regions, which was higher than that in the low-risk regions, the remaining incidence rates were all higher than those in the normal-risk and low-risk regions.
- The normal-risk regions include 12 administrative regions: Zizhi, Shulin, Shenkeng, Banqiao, Luzhou, Taishan, Xinzhuang, Ruifang, Yingge, Jinshan, Sanchong, and Danshui. The incidence rates of the five risk factors for metabolic syndrome were 39.10% for high-density lipoprotein cholesterol, 20.85% for triglycerides, 34.10% for blood sugar, 54.80% for blood pressure, and 34.64% for abdominal circumference; the overall incidence rate was 31.11%. Other than the incidence rate for high-density lipoprotein cholesterol in these regions, which was higher than that in the slightly high-risk and low-risk regions, the remaining incidence rates were all higher than those in the low-risk regions. The incidence rate was close to the mean value (30.63%).
- The low-risk regions include six administrative regions: Yonghe, Tucheng, Xindian, Wanli, Zhonghe, and Sanzhi. The incidence rates of the five risk factors for metabolic syndrome were 35.32% for high-density lipoprotein cholesterol, 18.93% for triglycerides, 30.34% for blood sugar, 49.06% for blood pressure, and 30.86% for abdominal circumference; the overall incidence rate was 25.66%. The incidence rates for the five risk factors in these regions were all lower than those in the other three groups.

We found that areas with higher incidence rates were mostly more remote regions, and among them, Wulai was found to have the highest incidence rate of metabolic syndrome. According to the statistics of medical facilities in New Taipei City, regions with more severe health problems have relatively fewer medical resources. Although the regional population numbers might be relatively lower, there are no large hospitals for patients to visit when more urgent situations are encountered, demonstrating the nonuniform distribution of hospitals in Taiwan. Next, using the survey information from heath screening programs, five lifestyle habits (smoking, consumption of alcohol, chewing betel nut, exercise, and regular health examinations) and eight dietary habits (consumption of milk, vegetables, three meals a day, desserts and snacks, fried food, and fats) were listed for a total of 13 factors. A decision tree analysis was performed to determine the influencing factors for each region within the cluster analysis. The importance of the factors in each region was obtained by sorting the frequency of the appearance of each factor, and the commonality of each group of factors was compared.

**Figure 3:**
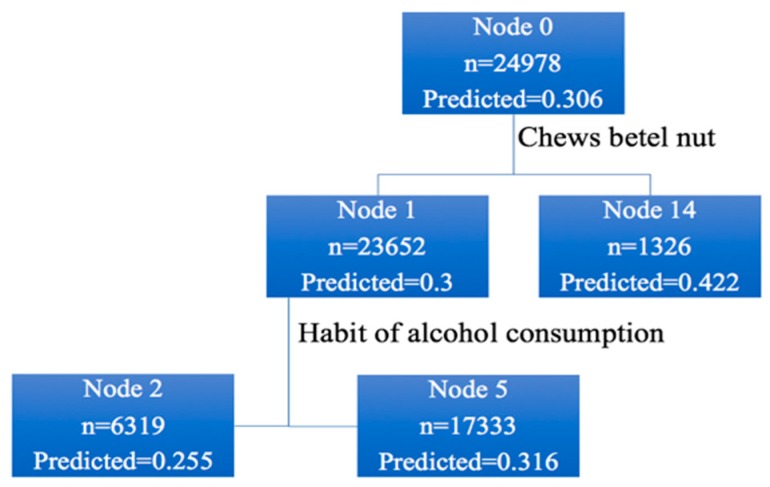
Decision tree analysis results.

In Figure 3, the total screening population was 24,978, with an incidence rate of 30.6%. Among these, 23,652 people did not have a habit of chewing betel nut (incidence rate, 30.0%). The number of people who had a habit of chewing betel nut or those who had quit chewing betel nut was 1326 (incidence rate, 42.2%). Among the 23,652 people who did not chew betel nut, 6319 quit alcohol (incidence rate, 25.5%) and 17,333 did not, or occasionally drank alcohol (incidence rate, 31.6%), and so on until there were no nodes to follow up.

Table 3 and Table 4 show the results for various groups after the decision tree analysis. The risk factors for metabolic syndrome in the high-risk region were chewing betel nut, consuming meat at every meal, consuming desserts or snacks regularly, smoking, frequently having lunch outside each week, frequently having dinner outside each week, drinking milk regularly, performing regular exercise, and the number of times breakfast was eaten each week. The risk factors for the slightly high-risk region were consuming alcohol, consuming desserts or snacks regularly, consuming fruits and vegetables, smoking, consuming fried or fat food more than three times a week, frequently having lunch outside each week, drinking milk regularly, chewing betel nut, consuming meat at every meal, having regular physical health examinations, and frequently having dinner outside each week. 

The risk factors for the normal-risk region were chewing betel nut, consumption of alcohol, frequently having lunch outside each week, smoking, and consuming desserts or snacks regularly. The risk factors for the low-risk regions were chewing betel nut, consuming alcohol, smoking, consuming desserts or snacks regularly, having regular physical health examinations, and regularly exercising. Overall, smoking, chewing betel nut, and consuming desserts or snacks regularly are crucial risk factors for metabolic syndrome in all the four groups.

## 4. Discussion

Previous studies on metabolic syndrome mostly use statistical methods to explore the incidence rates and risk factors or to find associations with diseases linked to metabolic syndrome. However, these studies did not investigate or compare metabolic syndrome specific to each administrative region. Do differences in geographical environment affect health? Dummer found an important association between geographical location and health status [13], and Hancock noted that factors affecting human health include health care organizations, human biology, environment, and lifestyle [14]. The occurrence of disease is related closely to age, sex, race, and lifestyle. Yu et al. [15] used the GIS package to determine the geographic differences in costs for chronic heart failure, diabetes, and spinal-cord injury. They found that analyses of geographic areas and health information can improve disease prevention and health plans and can effectively control the increasing cost of health care [16]. 

Due to the implementation of the health insurance system in Taiwan, it is necessary to develop strategies that are more effective and efficient for resource allocation, given that medical resources are declining every year. Therefore, it is necessary to determine the most urgent medical needs of various regions. For Taiwan, this information was not known in the past. Our study included people who participated in New Taipei City’s integrated community health screening service plan as research participants. Using technologies related to data exploration, we explored the incidence rates of metabolic syndrome in populations from various administrative regions and performed grouping and cause exploration to provide relevant departments with public health strategies specifically developed based on the differences in the administrative regions. In this study, the main differences with Taiwan’s past studies on metabolic syndrome are as follows: (1) the comprehensive community health check is free, so more information can be collected; (2) the severity of metabolic syndrome varies by region; (3) the risk factors for metabolic syndrome vary by region; (4) this understanding might help us more efficiently distribute medical resources to lower the proportion of people with metabolic syndrome.

Based on the grouping results from incidence rates, it was found that 11 administrative regions in New Taipei City, including Wulai, Shiding, Pinglin, Gongliao, Shimen, Sanxia, Wugu, Pingxi, Shuangxi, Linkou, and Bali, had a high incidence rate of metabolic syndrome. Among 3011 medical facilities in New Taipei City, there are only 150 medical facilities (five hospitals and 145 clinics) in the 11 administrative regions mentioned above. This shows the severe lack of medical resources in remote regions. We found that different disease patterns and health problems are experienced by inhabitants of different ages. This result is supported by Fond et al. [2], who found that the adult population of the US has a common metabolic syndrome, and that race, age, obesity, and lifestyle, including smoking and physical activity, affect its prevalence. Therefore, regarding health care and health promotion, the dietary and lifestyle habits of individuals should be improved.

## 5. Conclusions

Under the National Health Insurance system, Taiwan’s medical resources are limited. The results of this study can be an important reference for government agencies in planning medical resources. In addition, corresponding health care strategies for different regions can be proposed, which can effectively reduce and control metabolic syndrome in Taiwan. We propose suggestions for research, education, and health policy. In terms of research, the incidence rates of metabolic syndrome for people aged ≥ 40 years in New Taipei City was not low, and the incidence rates of metabolic syndrome among the sexes and age groups were significantly different. Additionally, the environment and region in which individuals are raised are also key factors that affect incidence rates. Lifestyles and dietary habits can vary by region, thus, affecting the incidence rate of metabolic syndrome. In terms of education, diseases associated with metabolic syndrome have become among the top ten causes of mortality in Taiwan. Moreover, national expenses regarding individual and family health are quite high. 

In the future, support is needed from local nongovernmental organizations to increase screening rates in each region, especially in the more remote regions, to identify more patients with metabolic syndrome. For preventive measures, we suggest public health interventions to develop good lifestyles and dietary habits, starting with young teenagers. In terms of health policy, the current integrated community health screening service targets people aged ≥40 years with free screening services. We suggest lowering the age for these screening services to promote earlier identification of patients with metabolic syndrome.

## Figures and Tables

**Table 1:** Determination standard for metabolic syndrome.

Risk Factors	Abnormal Values
Abdominal obesity	Waist circumference: males ≥90 cm (35.5 in); females ≥80 cm (31.5 in)
High blood pressure	Systolic pressure ≥130 mmHg; Diastolic pressure ≥85 mmHg
High fasting blood sugar level	≥100 mg/dL
High triglyceride level	≥150 mg/dL
Low high-density lipoprotein cholesterol level	Males, <40 mg/dL; Females, <50 mg/dL

**Table 2:** Analysis results of multivariate analysis of variance (MANOVA).

MANOVA	Two Groups	Three Groups	Four Groups
*p*-value	0.0	0.0	0.0
Pillai’s Trace	0.726	1.523	1.713

**Table 3:** Comparison of major risk factors for each group by lifestyle.

Lifestyle	High-Risk Region	Slightly High-Risk Region	Normal-Risk Region	Low-Risk Region
Smoking	●	●	●	●
Alcohol consumption		●	●	●
Chewing betel nut	●	●	●	●
Habit of exercising	●			●
Regular physical body examination		●		●

**Table 4:** Comparison of major risk factors for each group by dietary habits.

Dietary Habits	High-Risk Region	Slightly High-Risk Region	Normal-Risk Region	Low-Risk Region
Habit of consuming milk	●	●		
Intake of sufficient fruits and vegetables		●		
Number of times breakfast was eaten each week	●			
Number of times lunch was eaten outside each week	●	●	●	
Number of times dinner was eaten outside each week	●	●		
Habit of consuming desserts and snacks	●	●	●	●
Consumption of fried or fatty food more than three times a week		●		
Consumption of a lot of meat at every meal	●	●		
